# Minipuberty: Looking Back to Understand Moving Forward

**DOI:** 10.3389/fped.2020.612235

**Published:** 2021-01-18

**Authors:** Laura Lucaccioni, Viola Trevisani, Alessandra Boncompagni, Lucia Marrozzini, Alberto Berardi, Lorenzo Iughetti

**Affiliations:** ^1^Pediatric Unit, Department of Medical and Surgical Sciences of the Mothers, Children and Adults, University of Modena and Reggio Emilia, Modena, Italy; ^2^Department of Medical and Surgical Sciences of the Mothers, Children and Adults, Post Graduate School of Pediatrics, University of Modena and Reggio Emilia, Modena, Italy; ^3^Neonatal Intensive Care Unit, Department of Medical and Surgical Sciences of the Mothers, Children and Adults, University of Modena and Reggio Emilia, Modena, Italy

**Keywords:** minipuberty, neurobehavior, neonate, gonadotrophins, hypothalamic-pituitary-gonadal axis, hypogonadism

## Abstract

Hypothalamic-pituitary-gonadal (HPG) axis activation occurs three times in life: the first is during fetal life, and has a crucial role in sex determination, the second time is during the first postnatal months of life, and the third is with the onset of puberty. These windows of activation recall the three windows of the “Developmental Origin of Health and Disease” (DOHaD) paradigm and may play a substantial role in several aspects of human development, such as growth, behavior, and neurodevelopment. From the second trimester of pregnancy there is a peak in gonadotropin levels, followed by a decrease toward term and complete suppression at birth. This is due to the negative feedback of placental estrogens. Studies have shown that in this prenatal HPG axis activation, gonadotropin levels display a sex-related pattern which plays a crucial role in sex differentiation of internal and external genitalia. Soon after birth, there is a new increase in LH, FSH, and sex hormone concentrations, both in males and females, due to HPG re-activation. This postnatal activation is known as “minipuberty.” The HPG axis activity in infancy demonstrates a pulsatile pattern with hormone levels similar to those of true puberty. We review the studies on the changes of these hormones in infancy and their influence on several aspects of future development, from linear growth to fertility and neurobehavior.

## Introduction

During embryogenesis, the pituitary gland begins synthesizing both Luteinizing Hormone (LH) and Follicle-Stimulating Hormone (FSH) at around 9 weeks of gestation (1). LH and FSH can be detected in fetal blood from 12 to 14 weeks (2, 3) and start to be GnRH-dependent after 31–32 weeks (4, 5).

Prenatal modulation of the HPG axis activity is also due to placental hormone production. In fact, the structure of hCG is an analog of LH and may bind to the LH receptor, with similar biological effects on gonadal tissues (2, 6). Moreover, the placenta produces Estrogens (E) and Progesterone (P), that rise during the third trimester. This has a negative effect on gonadotropin levels and results in a drop in LH and FSH in cord blood at birth in healthy infants of both sexes (6–8).

After birth, the removal of placental hormones from the neonate's circulation results in a lack of negative feedback on the GnRH pulse generator and reactivation of the HPG axis. This postnatal activation that starts in the first few days of life is known as “minipuberty” (9).

Studies on healthy term neonates indicate that the rise of LH and FSH begins at around 1 week of age. It achieves a peak, reaching the pubertal range, between 1 and 3 months of life and then declines toward the age of 6 months (6–8, 10–13). These postnatal hormonal changes have different trends in boys and girls. Particularly in males, it seems to be related to the development and maturation of the reproductive system. Furthermore, the impact of gonadotropins and sex-steroid hormones during this first period of life has been studied and relates to many different aspects of infant growth and behavior (14).

The aim of this review is to summarize the current understanding on minipuberty and its role as a temporary window of opportunity for diagnosis and possible treatment in babies with disorders of sex development (DSD). Moreover, we would like to highlight the extent of what happens (or not) during minipuberty in terms of hormonal changes and trends which may influence future neurobehavior.

## Infants Born at Term

**Males:** In male neonates, both LH and FSH levels peak between 1 and 3 months of age and then gradually decrease to prepubertal levels at around 6-9 months (9, 12, 14). The LH peak is higher than the FSH level. Testosterone (T) starts to increase 1 week following the LH rise and declines to prepubertal values by 6 months of age (11, 12, 15). T levels, both in cord blood and in serum during the first postnatal months, are higher in boys than in girls (11–13, 15–17).

The number of Leydig cells in both testes increases considerably until the third month of life, which correlates with the T trend and then gradually decreases due to an apoptosis process (18, 19). Sertoli cells also grow during the first postnatal months under the stimulation of FSH (20) but, without the expression of androgen receptors (AR) during infancy, they do not complete their maturation and spermatogenesis does not occur (21). This leads to an increase of testicular volume during the first months after birth, which then gradually decreases until the second year of life due to the halt in cell proliferation, the reduction of AMH production, and the formation of the blood-testicular barrier (22, 23).

All these hormonal changes during the first months of life have a great impact on the urogenital system. This involves not only the testes but also the development and growth of the penis, prostate, and scrotal hair. In fact, the postnatal T surge within the first three months has been associated with penile growth in infancy (24). The increase in androgens has been associated with cutaneous manifestations, such as sebaceous gland hypertrophy and acne (25). There is also a link with the development of transient isolated scrotal hair between 3 and 6 months of life with a spontaneous disappearance within the first year of life (26).

**Females:** In female infants, FSH levels are higher than LH, following a different trend than in males. FSH shows the same gonadotropin peak as in males at 1–3 months of age but can remain elevated up to 3–4 years of life. In contrast, LH levels decrease at the same age as in boys (9, 12, 27).

E levels at birth are high, with similar values in the cord blood of both sexes (28), followed by a gradual decrease during the first days of life and a new increase after the first week only in girls.

E remains high until 6 months with fluctuating levels, probably related to the FSH trend, and decreases toward 2 years of life (29–31). The mammary glands and uterus are certainly E target tissues but evidence of minipuberty effects is not univocal. At birth, most full-term babies of both sexes have palpable breast tissue (32) that probably results from placental E effect. In the following months, breast tissue in females remains larger and persists longer due to HPG axis activity and its consequent E production (30). In contrast, uterine length increases *in utero* but, after birth, there is a steady decrease from day 7 toward the third month, after which the volume remains stable until the second year (30).

With little evidence from few studies, the biological role of minipuberty in girls is still controversial and partially unknown.

Babies **born small for gestational age (SGA):** The HPG axis activation in SGA infants born at term is not well defined and its short-term and long-term effects on growth and development are still controversial. Studies on SGA females found higher postnatal FSH levels compared with neonates born appropriate for gestational age (AGA). This different pattern of secretion in SGA females was also associated with reduced uterine and ovarian size that persisted into young adulthood (33, 34). Moreover, Anti Mullerian Hormone (AMH) levels have been reported to be higher in SGA girls at 2–3 months of life, suggesting possible altered follicular development (35).

In contrast with these findings, other studies have reported higher E in SGA females after the administration of a GnRH stimulation test, although the reported basal levels were not significantly different (35).

In male SGA term neonates, HPG axis activation has been linked both to lower (36) and higher (34) FSH and T (37) levels, with uncertain effects in adult life (38).

Further studies are necessary to clarify the pattern of minipuberty in SGA male and female infants, along with the clinical implications. It is important to bear in mind that SGA neonates are at increased risk of metabolic and endocrinological disorders. These include reduced insulin sensitivity and increased adrenal hyperandrogenism, with consequent precocious pubarche and reduced ovulation rate (39).

## Preterm Infants

Little is known about the influence of prematurity on HPG axis activity and its effects. Fewer studies have investigated this pattern longitudinally in preterm (PT) babies compared with those born full-term (FT). Preterm birth does not seem to influence the postnatal HPG axis activation, as gonadotropin levels begin to rise after birth (whenever that is, as fetal-placental interruption) with the same timing as in FT infants.

Moreover, this hormonal surge might be even stronger and more prolonged than in FT infants (40, 41). However, these data are not univocal (40–42) in either the amplitude or the duration between different sexes. Immaturity of the hypothalamic feedback has been suggested as a possible mechanism for this strong and prolonged activation, although its biological significance is still not completely understood.

**T**he most recent longitudinal data suggest that minipuberty declines at about the same post-term age in term neonates compared with premature infants, suggesting that the HPG activity is regulated in an evolutionarily way (13). In particular, Kuiri-Hanninen et al. (13) used spot urine samples in order to compare gonadotropin and testosterone levels in a small cohort of FT and PT male neonates with a gestational age (GA) between 24.7 and 36.6 weeks. From day 7 to 14 months of age they measured length, weight, penile length, and testicular volume. They simultaneously collected urine samples to detect urinary gonadotropins and testosterone levels until 6 months of age. Their findings revealed higher hormonal levels in PT babies with a positive association between testosterone levels and penile growth, as well as between FSH levels and testicular growth after birth until 5 months of age, when a subsequent decrease occurred. In addition, studies on PT female infants demonstrated higher gonadotropin levels than those in FT girls with a prolonged duration of the peak (43) but a sharp decrease around term age (30). In these PT girls, an amplified postnatal E surge was observed at around three months of corrected age and there was an association with increased growth of the mammary gland and uterine length. Possible clinical consequences of this intensive stimulation in premature females are evidenced by features of the ovarian hyperstimulation syndrome with edema of the vulva, solitary or multiple cysts in the ovaries on ultrasonography, breast growth, and occasional vaginal bleeding (44, 45).

The hormonal differences between boys and girls during minipuberty appear to be fundamental for later sexual differentiation and development. In particular, we think that increasing knowledge on minipuberty in girls may give us key information about the premature thelarche of girls below 2 years of age, and the early puberty that occurs before 8 years of age. In SGA neonates, results are still controversial and more studies are needed to clarify how gonadotrophins and sexual hormones change according to sex. This may be very useful, considering that SGA-born children may go through early puberty and/or precocious isolated pubarche. In preterm babies, we speculated that the prolonged activation of HPG axis may be one of the factors influencing the early re-activation of the HPG axis before puberty age.

## Babies With Disorders of Sex Development

The development of internal and external genitalia is a complex balance between gene expression and hormonal influence and an anomaly at each stage can result in 46, XY DSD.

From this point of view, minipuberty can be considered as a window of sensitivity, because it may allow the clinician to come to an early diagnosis and possible treatment opportunity.

Studies on primates testing the effects of a reversible suppression of minipuberty using GnRH agonists or antagonists described lower testis volume and penile length in cases treated compared with controls (46–48). Male infants with congenital central hypogonadism (CHH) were found to have an absence of both fetal and postnatal FSH, LH, and T surges (49). This lack of postnatal FSH secretion seems to be the main reason for impaired germ cell differentiation with later infertility, especially if associated with cryptorchidism (50, 51). As a result, minipuberty may potentially provide a short window of time to make an early diagnosis and for treatment in male neonates that exhibit a micropenis with or without cryptorchidism (52, 53), improving the outcome of orchidopexy, and also reducing the long-term consequences of an absent minipuberty.

On the other hand, the finding of elevated gonadotropins during minipuberty in a 46, XY male neonate with undetectable testosterone levels may suggest congenital anorchism (vanishing testis or testicular regression syndrome). Infants with complete androgen insensitivity syndrome may present with lower-than-normal postnatal LH and T levels, whereas these hormones may be normal or high in cases of partial androgen insensitivity syndrome (54).

We have a unique opportunity to evaluate the spontaneous function of the HPG hormone axis during minipuberty. It is therefore recommended that serum FSH, LH, and testosterone are measured during the first 6 months of life in infants with DSD or suspected CHH. In particular, we would suggest checking for these hormones at seven days of life, and one, three, and, if possible, 6 months of life, to detect the minipuberty trend. The use of the LH/FSH ratio may provide important information in the workup of infants suspected of DSD, especially regarding the sex specific ratio detected in literature (55).

## Minipuberty: A Window for Treatment?

Neonates affected by cryptorchidism, micropenis, and CHH must receive timely treatment to optimize genital development. The current recommendation for a micropenis is brief therapy with low-dose testosterone delivered by intramuscular injection or by topical application to induce penile growth. This treatment was also assumed to work for cryptorchidism. However, while exogenous T stimulates penile growth, it does not affect testicular development. In fact, current recommendations advocate surgical correction for undescended testes during the first year of life (56). Nevertheless, there are limitations to this treatment. Small testes augment the risk of testicular trauma and tissue loss during orchidopexy (57), increasing the likelihood of a negative impact on future fertility. Moreover, successful scrotal repositioning of testes does not prevent infertility. However, a normal minipuberty after successful surgery may lead to the presence of Ad spermatogonia (58). The role of Ad spermatogonia is to maintain the supply of stem cells for spermatogenesis. In 178 testicular biopsies after orchidopexy the authors found three groups of high, intermediate, and low risk of infertility depending on the presence of Ad spermatogonia. After puberty, sperm concentrations were analyzed and correlated positively with plasma gonadotropin and testosterone levels. For all these reasons, recreating the hormonal milieu of minipuberty with gonadotrophin treatment could be beneficial for these patients.

In 2002, Main et al. (59) published the first case of CHH and micropenis treated with short-term recombinant human LH and FSH. The outcome of this case was successful; the penile length increased by 50% and the testicular volume almost tripled. Similar results were described in other recent cases (60, 61).

The REMAP study (62) investigated the use of recombinant LH plus FSH preparations in neonates and infants with a micropenis and/or cryptorchidism due to hypogonadotropic hypogonadism. During therapy, all ten patients increased their height velocity: LH levels increased from undetectable to high-normal; FSH reached supranormal levels; and Inibin-b, AMH, and T reached normal levels. Penile length normalized among all children and intriguingly confirms the emerging evidence that testicular descendance is induced by gonadotrophin treatment (61, 63). Furthermore, in this study the therapy may have induced high/normal activation of Sertoli and Leydig cells, restoring testicular endocrine function and improving future fertility.

Vincel et al. (64) analyzed testicular biopsies before and after orchidopexy or hormonal treatment in patients with isolated bilateral cryptorchidism with a high infertility risk. Their results showed how the number of Ad spermatogonia and the number of germ cells per at least 100 tubular cross-sections increased or decreased post-surgery. Indeed, patients who received hormonal treatment showed an important increase in the number of cells and the complete transition of gonocyte and fetal spermatogonia to Ad spermatogonia. These findings support the hypothesis that GnRH induces LH release; LH increases testosterone levels acting directly on Leyding cells, mimicking minipuberty (50, 65).

Finally, studies have focused on the molecular mechanisms that explain the ability of GnRH to rescue fertility. The analysis demonstrates that several IncRNAs involved in epigenetic programming were responsive to GnRH treatment, helping in the preparation of Ad spermatogonial stem cells for commitment to differentiation. In particular, the authors found that DMRTC2, PAX7, BRACHYURY/T, and TERT were associated with defective minipuberty and were responsive to GnRHa (66). Minipuberty may represent a “window of opportunity” to evaluate the HPG axis by measuring basal hormone concentrations with no need for stimulation tests in infants with suspected reproductive disorders. Minipuberty provides a unique opportunity to evaluate the spontaneous function of the HPG axis which is lost thereafter for approximately another 10 years until the HPG axis is reactivated in puberty (67).

## Is Our Environment Influencing Minipuberty in Humans and Predisposing Them to DSD?

Endocrine Disruptor Chemicals (EDC) are compounds detectable in every setting of daily life. These chemical compounds are found in a range of products such as those containing pesticides, metals, additives or food contaminants, and personal care products (68). In fact, EDCs are so common that it is almost impossible for individuals to avoid them during everyday activities. These substances may cause adverse health effects, disrupting endocrine function. In particular, they interfere with the endocrine and reproductive systems through nuclear receptors, non-nuclear steroid hormone receptors, non-steroid receptors, orphan receptors, enzymatic pathways, and other mechanisms (69). Children may be exposed both directly and indirectly to ECDs, especially during the three main temporal windows of the DOHaD paradigm and during breastfeeding (70, 71). Concerning breastfed children, Ortega-Garcia et al. detected a linear positive correlation between anogenital distance (AGD) in male infants and the duration of breastfeeding (72). The results of this study, called MALAMA, suggested breastfeeding to be a protective factor against the reduction of the AGD of 2-year-old boys. The authors hypothesized this could be related to early exposure to EDCs through baby formula milk (72).

Moreover, EDCs may interfere with HPG activation (73), both during fetal life or immediately after birth, throughout minipuberty. EDCs during minipuberty in males could impair testicular descent (74). Focusing on some of the most analyzed compounds in this research area, several studies have demonstrated that Bisphenol A (BPA) has an anti-androgen function, decreasing testosterone levels, an event that impacts sex differentiation during fetal life and modifies the AGD length (75, 76). In particular, Sun et al. showed how maternal exposure to BPA was associated with shortened AGD in boys at 12 months of age, highlighting a gender specific effect (77). Another family of EDCs influencing minipuberty are phthalates. Maternal exposure to phthalates during pregnancy showed a reduced T level in males at minipuberty and, because of the antiandrogenic effect of these compounds, the testosterone-luteinizing hormone ratio (T/LH) is also lower in the same period (78). This is probably due to compensated Leydig cell function, requiring higher levels of LH to maintain the necessary level of T for embryo differentiation. It was demonstrated in animal models that phthalates can inhibit Insl3 production and consequently modify the gubernaculum growth necessary for the testes' transabdominal descent (79, 80). However, the effects of these compounds on Insl3 and T in the human testes were less attenuated than in rodents. Table 1 summarizes the most recent studies on the effect of EDC on HPG axis during minipuberty in humans. The impact of EDCs is not limited to the postnatal period. Indeed, alongside this phase there are two more windows of development: fetal and puberty. In these phases, cells are promptly proliferating, and epigenetic changes are more likely to occur (81). All this may lead to additional effects in later stages of life including delayed or precocious puberty (82–85), small testes and high levels of follicle-stimulating hormone (FSH) (86, 87), polycystic ovary syndrome (88), and breast cancer (89).

**Table 1:** Possible effects in humans of the main EDCs on minipuberty and long-term consequences (68–89).

**EDcs**	**Possible mechanism of action**	**Effects on minipuberty**	**Future effects**
BPA	Increased estrogen receptor Inhibition of apoptotic activity in breast tissue	Lower T level Shortened AGD in boys	Premature thelarche Breast neoplastic transformation Infertility
Phthalates	Reduced T synthesis Modified estrogen activity Antiandrogenic effect Insl3 inhibition	Lower T/LH ratio Undescended testis Hypospadias Shortened AGD	Early puberty Premature thelarche Delayed pubic hairs development Increased breast cells proliferation Less recruitment of primary follicles PCOS Spermatogenic failure and infertility
PBDEs/PBB	Modified estrogen activity Antiandrogenic effect	Undescended testis	Early pubic hair stage (boys) Early/late menarche in breastfed girls Early puberty Anticipated menarche
DDT/DDE	Modified estrogen expression Antiandrogenic effect	Undescended testis Hypospadias	Precocious puberty Anticipated menarche Later onset of puberty Increased risk of breast cancer Testicular cancer
PCBs	Augmented level of FSH and estradiol Antiandrogenic effect	Undescended testis Hypospadias	Anticipated menarche Delayed puberty Augmented adipose tissue in breast Semen alteration

Our environment plays a crucial role in developmental programming. The influence of EDCs on minipuberty may predispose an individual to undescended testis, AGD modifications, or reduction of T surge. We should always keep an eye on the appearance of the external genitalia in neonates and on the possible maternal exposure to phthalates and BPA through a specific interview.

## How Minipuberty Influences Linear Growth During the First 6 Months of Life

During minipuberty, the transient HPG axis activation results in a sex steroid surge. Some studies have indicated a higher growth velocity and a faster increase in weight (and lean body mass) associated with somatic changes in boys when compared with girls during the first 6 months of life (90–93). Based on these results, studies have tested the hypothesis of an association with minipuberty, particularly with the peak of testosterone production. Kiviranta et al. (94) evaluated the precise timing and the magnitude of this sexual dimorphism in growth among a large cohort of full-term healthy boys and girls during the first years of life. In a smaller sample of healthy neonates, serial measurements of urinary and blood hormones were assessed. Results from this study demonstrated that linear growth was significantly faster in boys than in girls, especially when comparing the first three months of age. Interestingly, this observation occurred simultaneously with the peak of postnatal gonadal activation and the authors found a positive correlation between T levels and growth velocity in both sexes, elucidating a possible novel biological role of minipuberty as an engine of growth velocity during the first months of life. Differences in sex hormones during minipuberty between boys and girls are important for the sex differentiation in linear growth and body composition, with males having a higher growth velocity and accumulating more lean mass compared to females.

## How Minipuberty Modulates Neurobehavioral Development

As for sexual development, the human brain is also shaped by a combination of genetic, epigenetic, environmental, and hormonal exposure. Sex steroid hormones are among one of the strongest biological factors influencing neural and behavioral development. Over the past two decades, there has been a growing interest in understanding how sex determination and sexual hormones may affect structural and functional brain development (95, 96). The cellular and molecular mechanisms induced by T (converted to estradiol in the brain) are multifaceted and include neurogenesis, cellular differentiation, axon guidance, synaptic pruning, apoptosis, and phagocytosis. Several studies on mammalian brains, including humans, have demonstrated that early androgen exposure has an influence on sex differences in juvenile behavior (54, 96, 97). Indeed, manipulating androgens prenatally in non-human primates alters brain regions and behaviors (98). Many studies have been performed in girls prenatally exposed to high levels of androgens because of congenital adrenal hyperplasia (CAH) where there is strong evidence of male typical play behavior, suggesting a similar hormonal influence on human brain development (98–104). This influence of androgen levels on the brain has been identified not only among affected girls but also in the general population. Fetal T measured from amniotic fluid positively correlates with male typical play in preschool girls and boys assessed with a standardized questionnaire (105). This prenatal period of HPG axis activation is therefore critical for the sexual differentiation that drives the different organization in circuitry and neuroanatomy between the male and female brain. The early postnatal surge of gonadotropins and T in boys during minipuberty can potentially provide a window of opportunity in understanding the effects of sex steroid hormones on human gender development (106). Emerging evidence suggests that T levels during minipuberty have an influence not only on male genitalia and reproductive function but also on later gender-typical behavior. Indeed, minipuberty occurs during a period of huge and rapid brain development in terms of volume, cortical thickness, surface, and cortical network development (107–109). Lamminmäki et al. (110) found a positive correlation between T levels in FT infants from day 7 to 6 months and future sex-typed behavior at 14 months of life. In this study, the Pre-School Activities Inventory (PSAI) (111, 112) playroom was used during an observation of toy choices. In boys, T levels correlated significantly with PSAI scores and playing with trains. Conversely, playing with dolls was significantly correlated with a negative trend. In addition, Pasterski et al. (113) used AGD at birth and penile growth from birth to 3 months of age to estimate prenatal and postnatal androgen exposure. They re-evaluated children included in the study at 3 to 4 years of age using the PSAI suggesting that T levels in both periods, prenatal and postnatal, are independent contributors to later gender-related behavior. Language development is another area of investigation of a possible correlation with early postnatal HPG axis activation. Results based on small samples suggest a correlation between T levels and a different expressive vocabulary in boys and girls (114, 115). We have summarized some of the clearest studies of the last decade in Table 2 in order to better understand the influence of hormonal changes happening during minipuberty on sex-related behavior. All these emerging results may support a role for the imprinting of T during early infancy in human neurobehavioral sexual differentiation, although its effects are still largely unknown in both the short and long-term.

**Table 2:** Main studies on minipuberty and neurobehavior.

**References**	**Methods**	**Results**	**Future prospectives**
Lamminmäki et al. (110)	– **Urinary testosterone** at 7 days of age (D7), and months 1, 2, 3, 4, 5 and 6 (M1–M6) – The **PSAI** is a 24-item, standardized questionnaire designed to discriminate gender related behavior within the sexes, as well as between girls and boys (111). It has been validated in the age-group 2 to 7 years. The questions covers three aspects of behavior: ***play with sex-typed toys*** (e.g., dolls, cars), ***engagement in sex-typed activities*** (e.g., ballgames, playing at cooking/cleaning) ***and sex-typed child characteristics*** (e.g., interest in snakes/spiders/insects, liking pretty things). – **The toy preference test**: the child was seated in the middle of a semi-circle formed by 9 toys. These toys were selected to be ***female- preferred*** (a tea set, a soft doll, and a baby doll and bathtub), ***male-preferred*** (a truck, a train, and a parking structure with two motorcycles), or ***gender neutral*** (a teddy bear, a soft picture book, and a set of keys). Two toys from the same category were never adjacent to each other. The session of 10 min was videotaped and the score consisted on how long (in seconds) that the child played with each toy, with play defined as the child touching the toy for 1 s or longer.	– In boys, **urinary testosterone** concentrations peaked at 1 month postnatal and decreased to low levels by the age 6 months. In girls, urinary testosterone concentrations were slightly elevated at D7 and M1, and then decreased to low levels. In the overall population, urinary testosterone was significantly higher in boys than in girls. – In boys, but not in girls, testosterone AUC correlated positively with **PSAI** scores. – Both boys and girls played significantly more with the same sex-related toy. – Testosterone in boys was negatively related to the female-preferred toy playing, but not in girls. Testosterone in girls was positively correlated with the male-preferred toy playing. A significant negative association between testosterone and time spent playing with the truck and a significant positive association between testosterone and time spent playing with the soft book among boys was detected.	The study underlined how testosterone may exert organizational effects on neurobehavioral development during early infancy both in girls and in boys. The urinary sampling method could be easier to be used in neonates and infants.
Constantinescu et al. (116)	61 healthy infants (29 males, 32 females) and 59 mothers and 3 fathers. **Saliva samples** of **testosterone** when infants were 1–2.5 months of age, and **mental rotation** performance was assessed at 5–6 months of age. Mental rotation ability was assessed using the procedure developed by Constantinescu et al. (116). The stimuli were video representations of dynamic 3D objects, depicted in rotational movement around their vertical axis in 3D space.	Testosterone concentrations were significantly higher in boys than in girls at age 1–2.5 months. In contrast, at age 5–6 months, testosterone concentrations were significantly lower in both sexes than they were at the first visit. – male infants spent a significantly longer time looking at the novel stimulus than at the familiar one, and 65% of the male infants preferred the novel stimulus. – In contrast, female infants looked at the familiar and novel test stimuli about equally, and 46% of the female infants preferred the novel stimulus. These findings suggest that more male than female infants had developed an ability for mental rotation at 5–6 months of age. – The male infants' novelty preference was significantly greater than that of the female infants – Testosterone concentrations at 1–2.5 months of age correlated significantly with novelty preference scores on the 3D mental rotation task in 5- to 6-month-old boys but not in girls.	– Testosterone may have organizational influences on mental rotation performance – In girls, mental rotation performance at age 5–6 months correlated negatively with parents' traditional attitudes on gender. This finding suggests that parents could influence their daughters. “mental rotation abilities” beginning very early in life.
Kung et al. (114)	– -Saliva samples for testosterone between 1 and 3 months old. – -Between 18 and 30 months, all of the parent participants were invited to complete an online questionnaire assessing the children's expressive vocabulary size – -The toddler short form for vocabulary production from the **MacArthur Communicative Development Inventory [CDI;** (117)] is a parent-report measure designed to assess expressive vocabulary production in toddlers aged 16–30 months	– -boys had significantly higher concentrations of testosterone during mini-puberty and significantly lower CDI scores at age 18–30 months than girls – there was a significant negative correlation between concentrations of testosterone during mini-puberty and later CDI scores in boys – Differences were found between boys and girls in salivary testosterone at 1–3 months of age and in expressive vocabulary size at 18–30 months of age. A negative link between testosterone during mini-puberty and expressive vocabulary was found in boys, in girls, and in the entire sample. Results also showed that testosterone accounted for significant additional variance in expressive vocabulary, when other predictors, such as child's age at vocabulary assessment and paternal education were controlled, suggesting that the effects of testosterone are independent from those of other predictors – higher concentrations of salivary testosterone during the peak of mini-puberty at age 1–3 months predicted smaller expressive vocabulary at age 18–30 months in boys and in girls.	Similar future research might usefully assess the independent contributions of prenatal and postnatal androgen exposure to expressive vocabulary and to other aspects of development that also differ by sex.
Kung et al. (118)	Testosterone in saliva samples collected from children at 1 to 3 months of age (40 boys, 47 girls). When the children reached 18 to 30 months of age, parents completed the **Quantitative Checklist for Autism in Toddlers (Q-CHAT)**.	Boys had higher concentrations of testosterone postnatally and higher Q-CHAT scores than girls. However, testosterone did not correlate with Q-CHAT scores in boys, girls, or the entire sample. There is no relationship between testosterone exposure during mini-puberty and autistic traits.	This does not preclude effects of mini-puberty on other behaviors (see the gender-typed play behavior). Other studies have hypothesized a correlation between prenatal exposure to testosterone and autistic traits.
Tanja Kuiri-Ha nninen et al. (13)	Urinary gonadotropins and testosterone were measured in serial urine samples and compared with testicular and penile growth in preterm (PT) and full term (FT) neonates. Urinary prostate-specific antigen was measured as an androgen biomarker.	LH and testosterone levels were higher in PT boys than FT boys. Compared with FT boys, FSH levels were lower at day 7 but higher from month 1 to month 3 in PT boys. This was associated with significantly faster testicular and penile growth in PT boys compared with FT boys.	Postnatal HPG axis activation in infancy is increased in PT boys and associated with faster testicular and penile growth compared with FT boys. As mentioned in Table 1, there is a possible long-term consequence of hyperandrogenism in PT infant boys warrant further research.
Pasterski et al. (113)	– Penile length, Ano-genital-distance (AGD), and body length were among the growth parameters assessed as part of the larger baby growth study. Measurements were taken at birth, and at 3, 12, 18, and 24 months of age in typically developing infants. – Gender-related behavior was measured at 3 to 4 years of age using the Preschool Activities Inventory (PSAI).	AGD at birth and penile growth during the first three months postnatal independently predicted increased masculine/decreased feminine behavior in boys at 3 to 4 years of age. AGD at birth may be employed as a biomarker of prenatal androgen exposure, while penile growth during mini-puberty may reliably reflect variance in early postnatal androgen exposure.	Future research could use these biomarkers in large-scale population studies to further elucidate neurobehavioral effects of perinatal androgen exposure. Such large-scale investigations could also permit a prospective assessment of other factors known to influence variance in gender-related behavior, such as socialization and cognitive development, along with their interactions with early androgen exposure.

## Conclusion

Although further studies are needed, pre- and postnatal activation of the HPG axis could be considered an important window of prediction on how each newborn will grow and develop. Measurement of LH, FSH, and testosterone at 7 days, one and three months, and, when possible, six months, may help the clinician to better understand how minipuberty develops in different neonates. This will give us important information once the baby approaches puberty or when he or she shows impaired linear growth. Moreover, minipuberty must be considered a fundamental moment for possible therapeutic intervention in DSD. Therapeutic interventions may be able to change the natural history of some DSD or, at least, to improve prognosis in terms of fertility and quality of life.

## Author Contributions

LL, VT, ABo, and LM analyzed the existing literature and wrote the draft of the review. ABe and LI critically reviewed the manuscript. All authors read and approved the final version of the manuscript. All authors contributed to the article and approved the submitted version.

## Conflict of Interest

The authors declare that the research was conducted in the absence of any commercial or financial relationships that could be construed as a potential conflict of interest.

## References

[1] Hagen C McNeilly AS. The gonadotrophins and their subunits in foetal pituitary glands and circulation. J Steroid Biochem. (1977) 8:537–44. 10.1016/0022-4731(77)90259-X 599925

[2] Clements JA Reyes FI Winter JSD Faiman C. Studies on human sexual development. III. fetal pituitary and serum, and amniotic fluid concentrations of LH. CG, and FSH. J Clin Endocrinol Metab. (1976) 42:9–19. 10.1210/jcem-42-1-9 1249196

[3] Kaplan SL Grumbach MM. The onotogenesis of human foetal hormones. II. Luteinizing hormone (LH) and follicle stimulating hormone (FSH). Acta Endocrinologica. (1976) 81:808–29. 10.1530/acta.0.0810808 946570

[4] Guimiot F Chevrier L Dreux S Chevenne D Caraty A Delezoide AL. Negative fetal FSH/LH regulation in late pregnancy is associated with declined kisspeptin/KISS1R expression in the tuberal hypothalamus. J Clin Endocrinol Metab. (2012) 97:E2221–9. 10.1210/jc.2012-2078 23015653

[5] Pilavdzic D Kovacs K Asa SL. Pituitary morphology in anencephalic human fetuses. Neuroendocrinology. (1997) 65:164–72. 10.1159/000127177 9087997

[6] Varvarigou AA Liatsis SG Vassilakos P Decavalas G Beratis NG. Effect of maternal smoking on cord blood estriol, placental lactogen, chorionic gonadotropin, FSH, LH, and cortisol. J Perin Med. (2009) 37:28. 10.1515/JPM.2009.028 19290844

[7] Winter JSD Faiman C Hobson WC Prasad AV Reyes FI. Pituitary-Gonadal relations in infancy. I. patterns of serum gonadotropin concentrations from birth to four years of age in man and chimpanzee. J Clin Endocrinol Metab. (1975) 40:545–51. 10.1210/jcem-40-4-545 1127071

[8] Winter JSD Hughes IA Reyes FI Faiman C. Pituitary-Gonadal relations in infancy: 2. Patterns of serum gonadal steroid concentrations in man from birth to two years of age. J Clin Endocrinol Metab. (1976) 42:679–86. 10.1210/jcem-42-4-679 1262442

[9] Kuiri-Hänninen T Sankilampi U Dunkel L. Activation of the hypothalamic-Pituitary-Gonadal axis in infancy: minipuberty. Horm Res Paediatr. (2014) 82:73–80. 10.1159/000362414 25012863

[10] Schmidt H Schwarz H. Serum concentrations of LH and FSH in the healthy newborn. Eur J Endocrinol. (2000) 2000:213–5. 10.1530/eje.0.1430213 10913940

[11] Bergadá I Milani C Bedecarrás P Andreone L Ropelato MG Gottlieb S. Time course of the serum gonadotropin surge, inhibins, and anti-Müllerian hormone in normal newborn males during the first month of life. J Clin Endocrinol Metab. (2006) 91:4092–8. 10.1210/jc.2006-1079 16849404

[12] Andersson A-M. Longitudinal reproductive hormone profiles in infants: peak of inhibin b Levels in infant boys exceeds levels in adult men. J Clin Endocrinol Metab. (1998) 83:675–81. 10.1210/jc.83.2.675 9467591

[13] Kuiri-Hänninen T Seuri R Tyrväinen E Turpeinen U Hamalainen E Stenman U-H. Increased activity of the hypothalamic-Pituitary-Testicular axis in infancy results in increased androgen action in premature boys. J Clin Endocrinol Metab. (2011) 96:98–105. 10.1210/jc.2010-1359 20881260

[14] Zegher FD Devlieger H Veldhuis JD. Pulsatile and sexually dimorphic secretion of luteinizing hormone in the human infant on the day of birth. Pediatr Res. (1992) 32:605–7. 10.1203/00006450-199211000-00025 1480465

[15] Forest MG Cathiard AM Bertrand JA. Evidence of testicular activity in early infancy. J Clin Endocrinol Metab. (1973) 37:148–51. 10.1210/jcem-37-1-148 4715291

[16] Barry JA Hardiman PJ Siddiqui MR Thomas M. Meta-analysis of sex difference in testosterone levels in umbilical cord blood. J Obst Gyn. (2011) 31:697–702. 10.3109/01443615.2011.614971 22085057

[17] Garagorri JM Rodríguez G Lario-Elboj ÁJ Olivares JL Lario-Muñoz Á Orden I. Reference levels for 17-hydroxyprogesterone, 11-desoxycortisol, cortisol, testosterone, dehydroepiandrosterone sulfate and androstenedione in infants from birth to six months of age. Eur J Pediatr. (2008) 167:647–53. 10.1007/s00431-007-0565-1 17694323

[18] Nistal M Paniagua R Regadera J Santamaria L Amat P. A quantitative morphological study of human leydig cells from birth to adulthood. Cell Tissue Res. (1986) 246:229–36. 10.1007/BF00215884 3779804

[19] Chemes HE. Infancy is not a quiescent period of testicular development. Int J Androl. (2001) 24:2–7. 10.1046/j.1365-2605.2001.00260.x 11168644

[20] Cortes D Müller J Skakkebæk NE. Proliferation of sertoli cells during development of the human testis assessed by stereological methods. Int J Androl. (1987) 10:589–96. 10.1111/j.1365-2605.1987.tb00358.x 3654012

[21] Chemes HE Rey RA Nistal M Regadera J Musse M Gonzàles-Peramato P. Physiological androgen insensitivity of the fetal, neonatal, and early infantile testis is explained by the ontogeny of the androgen receptor expression in sertoli cells. J Clin Endocrinol Metab. (2008) 93:4408–12. 10.1210/jc.2008-0915 18713818

[22] Kuijper EAM van Kooten J Verbeke JIML van Rooijen M Lambalk CB. Ultrasonographically measured testicular volumes in 0- to 6-year-old boys. Hum Reprod. (2008) 23:792–6. 10.1093/humrep/den021 18281246

[23] Cassorla FG Golden SM Johnsonbaugh RE Heroman WM Loriaux DL Sherins RJ. Testicular volume during early infancy. J Pediat. (1981) 99:742–3. 10.1016/S0022-3476(81)80398-8 7299549

[24] Boas M Boisen KA Virtanen HE Kaleva M Suomi AM Schmidt IM. Postnatal penile length and growth rate correlate to serum testosterone levels: a longitudinal study of 1962 normal boys. Eur J Endocrinol. (2006) 154:125–9. 10.1530/eje.1.02066 16382001

[25] Kuiri-Hänninen T Haanpää M Turpeinen U Hämäläinen E Dunkel L Sankilampi U. Transient postnatal secretion of androgen hormones is associated with acne and sebaceous gland hypertrophy in early infancy. J Clin Endocrinol Metab. (2013) 98:199–206. 10.1210/jc.2012-2680 23144469

[26] Janus D Wojcik M Tyrawa K Starzyk J. Transient isolated scrotal hair development in infancy. Clin Pediatr. (2013) 52:628–32. 10.1177/0009922813480845 23482727

[27] Kuiri-Hänninen T Kallio S Seuri R Tyvairen E Liakka A Tapanainen J. Postnatal developmental changes in the pituitary-ovarian axis in preterm and term infant girls. J Clin Endocrinol Metab. (2011) 96:3432–9. 10.1210/jc.2011-1502 21900380

[28] Troisi R Potischman N Roberts J Siiteri P DAftary A Sims C. Associations of maternal and umbilical cord hormone concentrations with maternal, gestational and neonatal factors (United States). Cancer Causes Control. (2003) 14:347–55. 10.1023/a:1023934518975 12846366

[29] Schmidt IM Chellakooty M Haavisto A-M Boisen KA Damgaard IN Steendahl U. Gender difference in breast tissue size in infancy: correlation with serum estradiol. Pediatr Res. (2002) 52:682–6. 10.1203/00006450-200211000-00012 12409513

[30] Kuiri-Hänninen T Haanpää M Turpeinen U Hamalainen E Seuri R Tyrvainen E. Postnatal ovarian activation has effects in estrogen target tissues in infant girls. J Clin Endocrinol Metab. (2013) 98:4709–16. 10.1210/jc.2013-1677 24217908

[31] Chellakooty M Schmidt IM Haavisto AM Boisen KA Damgaard IN Petersen JH. Inhibin a, inhibin b, follicle-Stimulating hormone, luteinizing hormone, estradiol, and sex hormone-Binding globulin levels in 473 healthy infant girls. J Clin Endocrinol Metab. (2003) 88:3515–20. 10.1210/jc.2002-021468 12915629

[32] Jayasinghe Y Cha R Horn-Ommen J O'Brien P Simmons PS. Establishment of normative data for the amount of breast tissue present in healthy children up to two years of age. J Pediat Adolesc Gynecol. (2010) 23:305–11. 10.1016/j.jpag.2010.03.002 20493740

[33] Ibáñez L Potau N Enriquez G Marcos MV de Zegher F. Hypergonadotrophinaemia with reduced uterine and ovarian size in women born small-for-gestational-age. Hum Reprod. (2003) 18:1565–9. 10.1093/humrep/deg351 12871863

[34] Ibáñez L Valls C Cols M Ferrer A Marcos MV de Zegher F. Hypersecretion of fSH in infant boys and girls born small for gestational age. J Clin Endocrinol Metab. (2002) 87:1986–8. 10.1210/jcem.87.5.8459 11994329

[35] Sir-Petermann T Hitchsfeld C Codner E Maliqueo M Iniguez G Echiburù B. Gonadal function in low birth weight infants: a Pilot study. J Pediat Endocrinol Metab. (2007) 20:405. 10.1515/JPEM.2007.20.3.405 17451079

[36] Nagai S Kawai M Myowa-Yamakoshi M Morimoto T Matsukura T Heike T. Gonadotropin levels in urine during early postnatal period in small for gestational age preterm male infants with fetal growth restriction. J Perinatol. (2017) 37:843–7. 10.1038/jp.2017.55 28448063

[37] Forest MG de Peretti E Bertrand J. Testicular and adrenal androgens and their binding to plasma proteins in the perinatal period: developmental patterns of plasma testosterone, 4-androstenedione, dehydroepiandrosterone and its sulfate in premature and small for date infants as compared with that of full-term infants. J Steroid Biochem. (1980) 12:25–36. 10.1016/0022-4731(80)90247-2 6448322

[38] Cicognani A Alessandroni R Pasini A Pirazzoli P Cassio A Barbieri E. Low birth weight for gestational age and subsequent male gonadal function. J Pediat. (2002) 141:376–80. 10.1067/mpd.2002.126300 12219058

[39] Ibáñez L de Zegher F Puberty after prenatal growth restraint. Horm Res. (2006) 65(Suppl. 3):112–5 10.1159/000091515 16612123

[40] Tapanainen J Koivisto M Vihko R Huhtaniemi I. Enhanced activity of the pituitary-Gonadal axis in premature human infants. J Clin Endocrinol Metab. (1981) 52:235–8. 10.1210/jcem-52-2-235 6780586

[41] Shinkawa O Furuhashi N Fukaya T Suzuki M Kono H Tachibana Y. Changes of serum gonadotropin levels and sex differences in premature and mature infant during neonatal life. J Clin Endocrinol Metab. (1983) 56:1327–31. 10.1210/jcem-56-6-1327 6404925

[42] Greaves RF Hunt RW Chiriano AS Zacharin MR. Luteinizing hormone and follicle-Stimulating hormone levels in extreme prematurity: development of reference intervals. Pediatrics. (2008) 121:e574–e80. 10.1542/peds.2007-1327 18310177

[43] de Jong M Rotteveel J Heijboer AC Cranendonk A Twisk JWR van Weissenbruch MM. Urine gonadotropin and estradiol levels in female very-low-birth-weight infants. Early Hum Dev. (2013) 89:131–5. 10.1016/j.earlhumdev.2012.09.007 23041221

[44] Sedin G Bergquist C Lindgren PG. Ovarian hyperstimulation syndrome in preterm infants. Pediatr Res. (1985) 19:548–52. 10.1203/00006450-198506000-00009 3892471

[45] Poon WYS Tung JYL. Vaginal bleeding in an infant with extreme prematurity. Case Rep Pediatr. (2020) 2020:8881634. 10.1155/2020/8881634 32802542 PMC7416300

[46] Lunn SF Cowen GM Fraser HM. Blockade of the neonatal increase in testosterone by a gnRH antagonist: the free androgen index, reproductive capacity and postmortem findings in the male marmoset monkey. J Endocrinol. (1997) 154:125–31. 10.1677/joe.0.1540125 9246946

[47] Nevison B Dixson F. Manipulation of postnatal testosterone levels affects phallic and clitoral development in infant rhesus monkeys. Int J Androl. (1999) 22:119–28. 10.1046/j.1365-2605.1999.00158.x 10194644

[48] Liu L Cristiano AM Southers JL. Effects of pituitary-Testicular axis suppression in utero and during the early neonatal period with a long-Acting luteinizing hormone-Releasing hormone analog on genital development, somatic growth, and bone density in male cynomolgus monkeys in the first 6 months of life. J Clin Endocrinol Metab. (1991) 73:1038–43. 10.1210/jcem-73-5-1038 1939516

[49] Grumbach MM. A window of opportunity: the diagnosis of gonadotropin deficiency in the male infant 1. J Clin Endocrinol Metab. (2005) 90:3122–7. 10.1210/jc.2004-2465 15728198

[50] Hadziselimovic F Zivkovic D Bica DTG Emmons LR The importance of mini-puberty for fertility in cryptorchidism. J Urol. (2005) 174(4 Part 2):1536–9. 10.1097/01.ju.0000181506.97839.b0 16148647

[51] Hadziselimovic F Emmons LR Buser MW. A diminished postnatal surge of ad spermatogonia in cryptorchid infants is additional evidence for hypogonadotropic hypogonadism. Swiss Med Wkly. (2004) 134:381–4. 15340882 10.4414/smw.2004.10575

[52] Quinton R Mamoojee Y Jayasena CN Young J Howard S Dunkel L. Society for endocrinology UK guidance on the evaluation of suspected disorders of sexual development: emphasizing the opportunity to predict adolescent pubertal failure through a neonatal diagnosis of absent minipuberty. Clin Endocrinol. (2017) 86:305–6. 10.1111/cen.13257 27749014

[53] Dwyer AA Jayasena CN Quinton R. Congenital hypogonadotropic hypogonadism: implications of absent mini-puberty. Min Endocrinol. (2016) 2016:10 10.3389/fendo.2019.00353 27213784

[54] Bouvattier C Carel J-C Lecointre C David A Sultan C Berrand A-M. Postnatal changes of t, lH, and fSH in 46,XY infants with mutations in the AR gene. J Clin Endocrinol Metab. (2002) 87:29–32. 10.1210/jcem.87.1.7923 11788616

[55] Johannsen TH Main KM Ljubicic ML Jensen TK Andersen HR Andersen MS. Sex differences in reproductive hormones during mini-Puberty in infants with normal and disordered sex development. J Clin Endocrinol Metab. (2018) 103:3028–37. 10.1210/jc.2018-00482 29917083

[56] Kolon TF Herndon CDA Baker LA Baskin LS Baxter CG Cheng EY. Evaluation and treatment of cryptorchidism: aUA guideline. J Urol. (2014) 192:337–45 10.1016/j.juro.2014.05.005 24857650

[57] Bouvattier C Maione L Bouligand J Dodé C Guiochon-Mantel A Young J Neonatal gonadotropin therapy in male congenital hypogonadotropic hypogonadism. Nat Rev Endocrinol. (2012) 8:172–82 10.1038/nrendo.2011.164 22009162

[58] Hadziselimovic F Hoecht B. Testicular histology related to fertility outcome and postpubertal hormone status in cryptorchidism. Klinische Padiatrie. (2008) 220:302–7 10.1055/s-2007-993194 18401814

[59] Main KM Schmidt IM Toppari J Skakkebaek NE. Early postnatal treatment of hypogonadotropic hypogonadism with recombinant human FSH and LH. Eur J Endocrinol. (2002) 146:75–9 10.1530/eje.0.1460075 11751071

[60] Bougnères P François M Pantalone L Rodrigue D Bouvattier C Demesteere E. Effects of an early postnatal treatment of hypogonadotropic hypogonadism with a continuous subcutaneous infusion of recombinant follicle-stimulating hormone and luteinizing hotmone. J Clin Endocrinol Metab. (2008) 93:2202–5 10.1210/jc.2008-0121 18381569

[61] Stoupa A Samara-Boustani D Flechtner I Pinto G Jourdon I Gonzaléz-Briceno L. Efficacy and safety of continuous subcutaneous infusion of recombinant human gonadotropins for congenital micropenis during early infancy. Horm Res Paediatr. (2017) 87:103–10 10.1159/000454861 28081535

[62] Papadimitriop DT Chrysis D Nyktari G Zoupanos G Liakou E Papadimitriou A. Replacement of male mini-Puberty. J Endocr Society. (2019) 3:1275–82 10.1210/js.2019-00083 31240270 PMC6584110

[63] Lambert A Bougnères P. Growth and descent of the testes in infants with hypognadotropic hypogonadism receiving subcoutaneous gonadotropin infusion. Int J Pediatr Endocrinol. (2016) 2016:13 10.1186/s13633-016-0031-9 27379168 PMC4931699

[64] Vincel B Verkauskas G Bilius V Dasevicius D Malcius D Jones B. Gonadotropin-Releasing hormone agonist corrects defective mini-Puberty in boys with cryptorchidism: a Prospective randomized study. BioMed Research International. (2018) 46:51218 10.1155/2018/4651218 30065939 PMC6051297

[65] Zivkovic D Bica DTG Hadziselimovic F. Relationship between adult dark spermatogonia and secretory capacity of leydig cells in cryptorchidism. BJU Int. (2007) 100:1147–9. 10.1111/j.1464-410X.2007.07034.x 17662083

[66] Gegenschatz-Schmid K Verkauskas G Demougin P Bilius V Dasevicius D Stadler MB. DMRTC2, PAX7, BRACHYURU/T and tert are implicated in male germ cell development following curative hormone treatment for cryptorchidism-induced infertility. Genes. (2017) 8:267. 10.3390/genes8100267 29019938 PMC5664117

[67] Renault CH Aksglaede L Wøjdemann D Hansen AB Jensen RB Juul A. Minipuberty of human infancy - a window of opportunity to evaluate hypogonadism and differences of sex development? Ann Pediatr Endocrinol Metab. (2020) 25:84–91 10.6065/apem.2040094.047 32615687 PMC7336259

[68] https://www.endocrine.org/advocacy/position-statements/endocrine-disrupting-chemicals (Accessed September 19, 2020).

[69] Phillips KP Foster WG. Key developments in endocrine disrupter research and human health. J Toxicol Environ Health B Crit Rev. (2008) 11:322–44 10.1080/10937400701876194 18368559

[70] Watkins DJ Sánchez BN Téllez-Rojo MM Lee JM Mercado-García A Blank-Goldenberg C. Phthalate and bisphenol a exposure during in utero windows of susceptibility in relation to reproductive hormones and pubertal development in girls. Environ Res. (2017) 159:143–51 10.1016/j.envres.2017.07.051 28800472 PMC5623649

[71] Bowman JD Choudhury M Phthalates in neonatal health: friend or foe? J Dev Orig Health Dis. (2016) 7:652–64 10.1017/S2040174416000349 27374623

[72] Ortega-Garcia JA Olano-Soler HA Martinez-Alvarez A Campillo-Lopez F Gomariz-Penalver V Mendiola-Olivares J. Breastfeeding duration and anogenital distance in 2-year-old infants. Breastfeed Med. (2016) 1:350–5. 10.1089/bfm.2016.0034 27403626 PMC5335819

[73] Winneke G Ranft U Wittsiepe J Kasper-Sonnenberg M Furst P Kramer U. Behavioral sexual dimorphism in school-age children and early developmental exposure to dioxins and pCBs: a follow-up study of the duisburg cohort. Environ Health Perspect. (2014) 122:292–8. 10.1289/ehp.1306533 24273228 PMC3948031

[74] Ünüvar T Büyükgebiz A. Fetal and neonatal endocrine disruptors. J Clin Res Pediatr Endocrinol. (2012) 4:51–60 10.4274/Jcrpe.569 22672860 PMC3386773

[75] Liu C Xu X Zhang Y Li W Huo X. Associations between maternal phenolic exposure and cord sex hormones in male newborns. Hum. Reprod. (2016) 31:648–56. 10.1093/humrep/dev327 26724800

[76] Steckler T Wang J Bartol FF Roy SK Padmanabhan V. Fetal programming: prenatal testosterone treatment causes intrauterine growth retardation, reduces ovarian reserve and increases ovarian follicular recruitment. Endocrinology. (2015) 146:3185–93. 10.1210/en.2004-1444 15802500

[77] Sun X Li D Liang H Miao M Song X Wang Z. Maternal exposure to biphenol a and anogenital distance troughout infancy: a longitudinal study from Shangai, China. Environ Int. (2018) 121(Pt 1):269–75. 10.1016/j.envint.2018.08.055 30223203

[78] Muerköster A-P Frederiksen H Juul A Andersson A-M Jensen RC Glintborg D. Maternal phthalate exposure associated with decreased testosterone/LH ratio in male offspring during mini-puberty. Odense Child Cohort. Environ Int. (2020) 144:106025 10.1016/j.envint.2020.106025 32798799

[79] Howdeshell KL Furr J Lambright CR Rider CV Wilson VS Gray LE. Cumulative effects of dibutyl phthalate and diethylhexyl phthalate on male rat reproductive tract development: altered fetal steroid hormones and genes. Toxicological Sciences. (2007) 99:190–202. 10.1093/toxsci/kfm069 17400582

[80] Borch J Metzdorff SB Vinggaard AM Brokken L Dalgaard M. Mechanisms underlying the anti-androgenic effects of diethylhexyl phthalate in fetal rat testis. Toxicology. (2006) 223:144–55 10.1016/j.tox.2006.03.015 16690193

[81] Gore AC Chappell VA Fenton SE Flaws JA Nadal A Prins GS EDC-2: the endocrine society's second scientific statement on endocrine-Disrupting chemicals. Endocr Rev. (2015) 36:E1–E50 10.1210/er.2015-1010 PMC4702494 26544531

[82] Vasiliu O Muttineni J Karmaus W. *In utero* exposure to organochlorines and age at menarche. Hum Reprod. (2004) 19:1506–12 10.1093/humrep/deh292 15131079

[83] Windham GC Pinney SM Voss RW Sjödin A Biro FM Greenspan LC. Brominated flame retardants and other persistent organohalogenated compounds in relation to timing of puberty in a longitudinal study of girls. Environ Health Perspect. (2015) 123:1046–52 10.1289/ehp.1408778 25956003 PMC4590751

[84] Hashemipour M Kelishadi R Amin MM Ebrahim K. Is there any association between phthalate exposure and precocious puberty in girls? Environ Sci Pollut Res Int. (2018) 25:13589–96 10.1007/s11356-018-1567-4 29497942

[85] Chen Y Wang Y Ding G Tian Y Zhou Z Wang X. Association between bisphenol a exposure and idiopathic central precocious puberty (ICPP) among school-aged girls in shanghai, china. Environ Int. (2018) 115:410–6 10.1016/j.envint.2018.02.041 29650233

[86] Hsu PC Lai TJ Guo NW Lambert GH Leon Guo Y. Serum hormones in boys prenatally exposed to polychlorinated biphenyls and dibenzofurans. J Toxicol Environ Health A. (2005) 68:1447–56. 10.1080/15287390590967360 16076757

[87] Yang CY Yu ML Guo HR Lai TJ Hsu CC Lambert G. The endocrine and reproductive function of the female yucheng adolescents prenatally exposed to pCBs/PCDFs. Chemosphere. (2005) 61:355–6 10.1016/j.chemosphere.2005.02.089 15885742

[88] Palioura E Diamanti-Kandarakis E. Polycystic ovary syndrome (PCOS) and endocrine disrupting chemicals (EDCs). Rev Endocr and Met Disorders. (2016) 16:365–71 10.1007/s11154-016-9326-7 26825073

[89] Burks H Martin E Mclachlan J Bunnell B Burow M. Endocrine disruptors and the tumor microenvironment: a new paradigm in breast cancer biology. Mol Cell Endocrinol. (2017) 457:13–9 10.1016/j.mce.2016.12.010 28012841

[90] Roche AF Guo S Moore WM. Weight and recumbent length from 1 to 12 mo of age: reference data for 1-mo increments. Am J Clin Nutr. (1989) 49:599–607. 10.1093/ajcn/49.4.599 2929481

[91] Rosen O Cohen A. Analysis of growth curves via mixtures. Statist Med. (2003) 22:3641–54. 10.1002/sim.1582 14652866

[92] Gasser T Sheehy Al Molinari. Sex dimorphism in growth. Ann Hum Biol. (2000) 27:187–97. 10.1080/030144600282299 10768423

[93] Becker M Oehler K Partsch C-J Ulmen U Schmutzler R Cammann H. Hormonal 'minipuberty' influences the somatic development of boys but not of girls up to the age of 6 years. Clin Endocrinol. (2015) 83:694–701. 10.1111/cen.12827 26031777

[94] Bizzarri C Cappa M. Ontogeny of hypotalamus-Pituitary gonadal axis and minipuberty: an ongoing debate? Front Endocrinol. (2020) 11:187 10.3389/fendo.2020.00187 32318025 PMC7154076

[95] Kiviranta P Kuiri-Hanninen T Saari A Lamidi M-L Dunkel L Sankilampi U. Transient postnatal gonadal activation and growth velocity in infancy. Pediatrics. (2016) 138:e20153561–e20153561. 10.1542/peds.2015-3561 27283013

[96] Lenroot RK Gogtay N Greenstein DK Molloy Wells E Wallace GL Clasen LS. Sexual dimorphism of brain developmental trajectories during childhood and adolescence. NeuroImage. (2007) 36:1065–73. 10.1016/j.neuroimage.2007.03.053 17513132 PMC2040300

[97] Goddings A-L Beltz A Peper JS Crone EA Braams BR. Understanding the role of puberty in structural and functional development of the adolescent brain. J Res Adolesc. (2019) 29:32–53. 10.1111/jora.12408 30869842

[98] Hines M Brook C Conway GS. Androgen and psychosexual development: core gender identity, sexual orientation, and recalled childhood gender role behavior in women and men with congenital adrenal hyperplasia (CAH). J Sex Res. (2004) 41:75–81. 10.1080/00224490409552215 15216426

[99] Ehrhardt A Meyer-Bahlburg H. Effects of prenatal sex hormones on gender-related behavior. Science. (1981) 211:1312–8. 10.1126/science.7209510 7209510

[100] New MI. Diagnosis and management of congenital adrenal hyperplasia. Annu Rev Med. (1998) 49:311–28. 10.1146/annurev.med.49.1.311 9509266

[101] Berenbaum SA Hines M Early androgens are related to childhood sex-Typed toy preferences. Psychol Sci. (1992) 3:203–6. 10.1111/j.1467-9280.1992.tb00028.x

[102] Pasterski VL Geffner ME Brain C Hindmarsh P Brook C Hines M. Prenatal hormones and postnatal socialization by parents as determinants of male-typical toy play in girls with congenital adrenal hyperplasia. Child Dev. (2005) 76:264–78. 10.1111/j.1467-8624.2005.00843.x 15693771

[103] Pasterski V Zucker KJ Hindmarsh PC Hughes IA Acerini C Spencer D. Increased cross-Gender identification independent of gender role behavior in girls with congenital adrenal hyperplasia: results from a standardized assessment of 4- to 11-Year-Old children. Arch Sex Behav. (2015) 44:1363–75. 10.1007/s10508-014-0385-0 25239661

[104] Frisén L Nordenström A Falhammar H Flipsson H Holmdahl G Janson PO. Gender role behavior, sexuality, and psychosocial adaptation in women with congenital adrenal hyperplasia due to CYP21A2 deficiency. J Clin Endocrinol Metab. (2009) 94:3432–9. 10.1210/jc.2009-0636 19567521

[105] Auyeung B Baron-Cohen S Ashwin E Knickmeter R Taylor K Hackett G. Fetal testosterone predicts sexually differentiated childhood behavior in girls and in boys. Psychol Sci. (2009) 20:144–8. 10.1111/j.1467-9280.2009.02279.x 19175758 PMC2778233

[106] Hines M Spencer D Kung KT Browne WV Constantinescu M Noorderhaven RM The early postnatal period, mini-puberty, provides a window on the role of testosterone in human neurobehavioural development. Curr Opin Neurobiol. (2016) 38:69–73. 10.1016/j.conb.2016.02.008 26972372

[107] Knickmeyer RC Gouttard S Kang C Evans D Wilber K Smith JK. A structural MRI study of human brain development from birth to 2 years. J Neurosci. (2008) 28:12176–82. 10.1523/JNEUROSCI.3479-08.2008 19020011 PMC2884385

[108] Lyall AE Shi F Geng X Woolson S Wang L Hamer RM. Dynamic development of regional cortical thickness and surface area in early childhood. Cereb Cortex. (2015) 25:2204–12. 10.1093/cercor/bhu027 24591525 PMC4506327

[109] Li G Lin W Gilmore JH Shen D. Spatial patterns, longitudinal development, and hemispheric asymmetries of cortical thickness in infants from birth to 2 years of age. J Neurosci. (2015) 35:9150–62. 10.1523/JNEUROSCI.4107-14.2015 26085637 PMC4469740

[110] Lamminmäki A Hines M Kuiri-Hänninen T Kilpeläinen L Dunkel L Sankilampi U. Testosterone measured in infancy predicts subsequent sex-typed behavior in boys and in girls. Horm Behav. (2012) 61:611–6. 10.1016/j.yhbeh.2012.02.013 22373494

[111] Golombok S Rust J. The measurement of gender role behaviour in pre-School children: a research note. J Child Psychol Psychiat. (1993) 34:805–11. 10.1111/j.1469-7610.1993.tb01072.x 8340446

[112] Golombok S Rust J Zervoulis K Croudace T Golding J Hines M. Developmental trajectories of sex-Typed behavior in boys and girls: a Longitudinal general population study of children aged 2.5-8 years. Child Dev. (2008) 79:1583–93. 10.1111/j.1467-8624.2008.01207.x 18826544

[113] Pasterski V Acerini CL Dunger DB Ong KK Hughes IA Thankamony A. Postnatal penile growth concurrent with mini-puberty predicts later sex-typed play behavior: evidence for neurobehavioral effects of the postnatal androgen surge in typically developing boys. Horm Behav. (2015) 69:98–105. 10.1016/j.yhbeh.2015.01.002 25597916

[114] Kung KTF Browne WV Constantinescu M Noorderhaven RM Hines M. Early postnatal testosterone predicts sex-related differences in early expressive vocabulary. Psychoneuroendocrinology. (2016) 68:111–6. 10.1016/j.psyneuen.2016.03.001 26970201

[115] Schaadt G Hesse V Friederici AD. Sex hormones in early infancy seem to predict aspects of later language development. Brain Lang. (2015) 141:70–6. 10.1016/j.bandl.2014.11.015 25540858

[116] Constantinescu M Moore DS Johnson SP Hines M. Early contributions to infants' mental rotation abilities. Dev Sci. (2018) 21:e12613. 10.1111/desc.12613 29143410

[117] Fenson L Bates E Dale P Goodman J Reznick JS Thal D. Measuring variability in early child language: don't shoot the messenger. Child Dev. (2000) 71:323–8. 10.1111/1467-8624.00147 10834467

[118] Kung KT Constantinescu M Browne WV Noorderhaven RM Hines M. No relationship between early postnatal testosterone concentrations and autistic traits in 18 to 30-month-old children. Mol Autism. (2016) 7:15. 10.1186/s13229-016-0078-8 26893820 PMC4757970

